# Prediction of breast cancer proteins involved in immunotherapy, metastasis, and RNA-binding using molecular descriptors and artificial neural networks

**DOI:** 10.1038/s41598-020-65584-y

**Published:** 2020-05-22

**Authors:** Andrés López-Cortés, Alejandro Cabrera-Andrade, José M. Vázquez-Naya, Alejandro Pazos, Humberto Gonzáles-Díaz, César Paz-y-Miño, Santiago Guerrero, Yunierkis Pérez-Castillo, Eduardo Tejera, Cristian R. Munteanu

**Affiliations:** 1 Centro de Investigación Genética y Genómica, Facultad de Ciencias de la Salud Eugenio Espejo, Universidad UTE, Mariscal Sucre Avenue, Quito, 170129 Ecuador; 2 RNASA-IMEDIR, Computer Science Faculty, University of Coruna, Coruna, 15071 Spain; 3 Red Latinoamericana de Implementación y Validación de Guías Clínicas Farmacogenómicas (RELIVAF-CYTED), Quito, Ecuador; 4 Grupo de Bio-Quimioinformática, Universidad de Las Américas, Avenue de los Granados, Quito, 170125 Ecuador; 5 Carrera de Enfermería, Facultad de Ciencias de la Salud, Universidad de Las Américas, Avenue de los Granados, Quito, 170125 Ecuador; 6 Centro de Investigación en Tecnologías de la Información y las Comunicaciones (CITIC), Campus de Elviña s/n 15071, A Coruña, Spain; 7 Biomedical Research Institute of A Coruña (INIBIC), University Hospital Complex of A Coruña (CHUAC), 15006 A Coruña, Spain; 8 Department of Organic Chemistry II, University of the Basque Country UPV/EHU, Leioa 48940, Biscay, Spain; 9 IKERBASQUE, Basque Foundation for Science, Bilbao 48011 Biscay, Spain; 10 Escuela de Ciencias Físicas y Matemáticas, Universidad de Las Américas, Avenue de los Granados, Quito, 170125 Ecuador; 11 Facultad de Ingeniería y Ciencias Agropecuarias, Universidad de Las Américas, Avenue de los Granados, Quito, 170125 Ecuador

**Keywords:** Breast cancer, Breast cancer

## Abstract

Breast cancer (BC) is a heterogeneous disease where genomic alterations, protein expression deregulation, signaling pathway alterations, hormone disruption, ethnicity and environmental determinants are involved. Due to the complexity of BC, the prediction of proteins involved in this disease is a trending topic in drug design. This work is proposing accurate prediction classifier for BC proteins using six sets of protein sequence descriptors and 13 machine-learning methods. After using a univariate feature selection for the mix of five descriptor families, the best classifier was obtained using multilayer perceptron method (artificial neural network) and 300 features. The performance of the model is demonstrated by the area under the receiver operating characteristics (AUROC) of 0.980 ± 0.0037, and accuracy of 0.936 ± 0.0056 (3-fold cross-validation). Regarding the prediction of 4,504 cancer-associated proteins using this model, the best ranked cancer immunotherapy proteins related to BC were RPS27, SUPT4H1, CLPSL2, POLR2K, RPL38, AKT3, CDK3, RPS20, RASL11A and UBTD1; the best ranked metastasis driver proteins related to BC were S100A9, DDA1, TXN, PRNP, RPS27, S100A14, S100A7, MAPK1, AGR3 and NDUFA13; and the best ranked RNA-binding proteins related to BC were S100A9, TXN, RPS27L, RPS27, RPS27A, RPL38, MRPL54, PPAN, RPS20 and CSRP1. This powerful model predicts several BC-related proteins that should be deeply studied to find new biomarkers and better therapeutic targets. Scripts can be downloaded at https://github.com/muntisa/neural-networks-for-breast-cancer-proteins.

## Introduction

The intricate interplay between several biological aspects such as environmental determinants, gene expression deregulation, genetic alterations, signaling pathway alterations and ethnicity causes the development of breast cancer (BC), a heterogeneous disease^1,2^. Over the last years, multi-omics studies, pharmacogenomics treatments and precision medicine strategies have evolved favorably; however, there are still biases such as the significant inclusion of minority populations in cancer research^3–7^. Nowadays, BC is the most commonly diagnosed cancer (2,088,849; 24% cases), and the leading cause of cancer-related deaths among women (626,679; 15% cases) worldwide^8^.

In our previous study, López-Cortés *et al*. developed the OncoOmics strategy to reveal essential genes in BC^9^. This strategy was a compendium of approaches that analyzed genomic alterations, protein expression, protein-protein interactome (PPi) network, dependency maps in cell lines and patient-derived xenografts of BC genes / proteins using relevant databases such as the Pan-Cancer Atlas project^3,10–12^, The Cancer Genome Atlas (TCGA)^13^, The Human Protein Atlas (HPA)^14–16^, the DepMap project^17–19^, and the OncoPPi network^20^.

Gene sets were taken from the Consensus Strategy^21^, the Pan-Cancer Atlas^3,11,12,22^, the Pharmacogenomics Knowledgebase (PharmGKB) ^23,24^, and the Cancer Genome Interpreter^25^. The Consensus Strategy, developed by López-Cortés *et al*., Tejera *et al*., and Cabrera-Andrade *et al*., was proved to be highly efficient in the recognition of genes associated with BC pathogenesis^21,26,27^. The Pan-Cancer Atlas reveals how genomic alterations, such as protein expression, copy number alterations (CNAs), mRNA expression, and putative mutations collaborate in BC progression^11,22,28–32^. PharmGKB is a comprehensive resource that collects the precise guidelines for the application of pharmacogenomics in clinical practice^23,24^. Lastly, the Cancer Genome Interpreter flags genomic biomarkers of drug response with different levels of clinical relevance^25^.

The OncoOmics BC essential genes were rationally filtered to 140. *RAC1*, *AKT1*, *CCND1*, *PIK3CA*, *ERBB2*, *CDH1*, *MAPK14*, *TP53*, *MAPK1*, *SRC*, *RAC3*, *BCL2*, *CTNNB1*, *EGFR*, *CDK2*, *GRB2*, *MED1*, and *GATA3* were significant in at least three OncoOmics approaches^9^. On the other hand, g:Profiler lets us know the enrichment map of the 140 essential genes in BC^33^. The most significant gene ontologies (GO) related to biological process and molecular function were the positive regulation of macromolecule metabolic process and the phosphatidylinositol 3-kinase activity, respectively. The most significant term, according to the Human Phenotype Ontology, was breast carcinoma^34^. Subsequently, the most relevant network interactions of the GO: biological process and the Reactome pathways were related to the immune system^35^, tyrosine kinase^36^, cell cycle^37^, DNA repair^38^, and RNA-binding proteins^39^. The Open Targets Platform has a largest number of drugs involved in clinical trials to treat BC with a direct focus on the OncoOmics BC essential genes were small molecules that correspond most likely to tyrosine kinases^40^. Hence, the essential proteins with signaling function are the interesting drug targets to modify any biological activity.

Starting a screening applying theoretical methods could save economic resources and time. Therefore, machine-learning (ML) techniques could obtain classification models that links signaling activity to protein structure. ML encodes molecular features into invariant descriptors based on physical and chemical properties of the amino acids, 3D protein conformation, graph topology, and protein sequences. The classification model is a quantitative structure-activity relationship (QSAR) between the biological function and the protein structure^41^. Different classification models have been published for prediction of protein activities: anti-oxidant^42^, lectins^43^, signaling^44^, anti-angiogenic^45^, anti-cancer^46^, and enzyme class^47^. Vilar *et al*. developed a QSAR model for alignment-free prediction of BC biomarkers using a linear discriminant analysis method, electrostatic potentials of protein pseudofolding HP-lattice networks as features, and 122 proteins related to BC and a control group of 200 proteins with classifications above 80%^48^. Our group proposed an improved multi-target classification model for human breast and colon cancer-related proteins by using a similar molecular graph theory for descriptors: star graph topological indices^49^. The accuracy of the models was 90.0% for a linear forward stepwise model. Both models presented linear relationships between graph-based protein sequence descriptors and BC, and unbalanced datasets. Thus, the aim of this study was to obtain an effective machine-learning classification model to predict BC-related proteins screening cancer immunotherapy proteins (CIPs), metastasis driver proteins (MDPs) and RNA-binding proteins (RBPs), using non-graph protein sequence descriptors and additional non-linear machine-learning techniques.

## Methods

**Figure 1:**
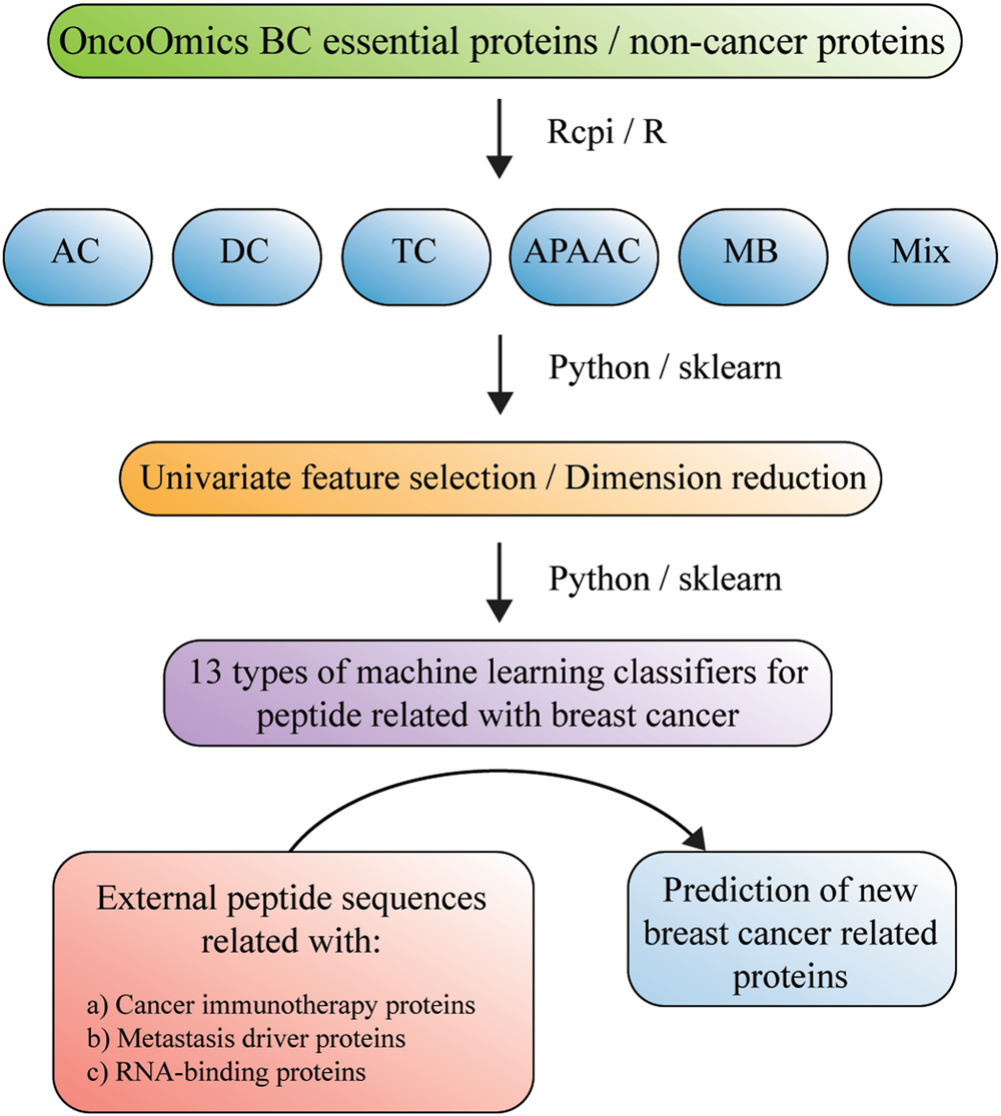
Flow chart of methodology for breast cancer (BC) protein prediction. AC, amino acid composition; DC, di-amino acid composition; TC, tri-amino acid composition; APAAC, amphiphilic pseudo-amino acid composition; MB, Moreau-Broto autocorrelation; Mix, total descriptors.

Figure 1 presents the general flow chart of the methodology to obtain a classifier for BC proteins. In the first step, we constructed a database with BC essential proteins and non-cancer proteins. In the second step, five families of Rcpi (R package)^50^ molecular descriptors have been used: 20 amino acid composition (AC), 400 di-amino acid composition (DC), 8000 tri-amino acid composition (TC), 80 amphiphilic pseudo-amino acid composition (APAAC), and 240 normalized Moreau-Broto autocorrelation (MB). The six sets of descriptors were constructed by mixing all the five-descriptor families, resulting 8,708 total descriptors (Mix).

Jupyter notebooks with python/sklearn^51^ were used to test 13 types of machine-learning classifiers for each set of descriptors, without feature selection, with univariate feature selection, or using principal component analysis (PCA)^52^. The classifiers were Gaussian Naive Bayes (NB)^53^, k-nearest neighbors algorithm (KNN)^54^, linear discriminant analysis (LDA)^55^, support vector machine (SVM) linear and non-linear based on radial basis functions (RBF), support vector classification (SVC) kernel = linear, and SVC kernel = RBF^56^, logistic regression (LR)^57^, multilayer perceptron (MLP) / neural network with 20 neurons in one hidden layer^58^, decision tree (DT)^59^, random forest (RF)^60^, XGBoost (XGB) is an optimized and distributed gradient boosting library^61^, Gradient Boosting for classification (GB)^62^, AdaBoost classifier (AdaB)^63^, and Bagging classifier (Bagging)^64^. The feature selection method was univariate filter such as SelectKBest (chi2, k), and the dimension reduction technique was PCA^52^.

Gaussian Naive Bayes is based on Bayes’ theorem and considers all the features are independent^53^. k-nearest neighbors algorithm assigns an unclassified sample using the nearest of k samples in the training set^54^. Linear discriminant analysis is a basic linear classifier^55^. SVM linear is using a higher dimensionality space to map the input features^56^. For non-linear problems, SVM uses Gaussian radial basis as non-linear kernels.

Logistics regression is another linear classifier that is able to calculate probability of a binary response using weights^57^. Multilayer perceptron represents a basic neural network with one hidden layer and with an ability to combine linear and nonlinear functions inside artificial neurons^58^. Decision tree represents a tree-type structure of decision rules obtained from the inputs^59^. Random forest is an ensemble method that combines parallel decision trees^60^. XGBoost uses sequential weak trees to improve the classification performance^61^. Gradient Boosting for classification is a basis boost method using sequential weak classifiers^62^. AdaBoost classifier is mixing different classifiers: it starts the fitting with a classifier based on the original dataset and adds additional copies of the original classifier with adjusted weights for the incorrectly classified instances^63^. Bagging classifier is a modified version of AdaB: the additional classifiers are based on subsets of the original dataset^64^.

The machine-learning prediction model was constructed from two protein sets. On the one hand, the positive set named OncoOmics BC essential proteins was made up of 140 strongly associated proteins to BC pathogenesis, according to López-Cortés *et al*.^9^. On the other hand, the negative protein set was constructed as follows: non-cancer proteins from Piazza *et al*.^65^, without BC-related proteins, were reanalyzed using Piazza’s OncoScore algorithm (http://www.galseq.com/oncoscore.html), giving a final list of 233 non-cancer proteins. Supplementary Tables 1 and 2 detail the sets and FASTA sequences of the OncoOmics BC essential proteins and the non-cancer proteins, respectively.

Three lists of cancer-related proteins were scanned with the final machine-learning prediction model: 1,232 CIPs were taken from Patel *et al*.,^35^ 1,903 MDPs were taken from the Human Cancer Metastasis Database (HCMDB) (http://hcmdb.i-sanger.com/index)^66^, and 1,369 RBPs were taken from Hentze *et al*.,^39^ (Supplementary Tables 3 to 5).

After the calculation of amino acid composition descriptors, the datasets contained 373 proteins. The BC class was labeled with 1 and non-cancer class with 0. Several preprocessing was done before any calculation: elimination of doubled examples, elimination of data with NA values, and elimination of features with zero variance. All feature values were normalized to values between 0 and 1 using MinMax() scaler. A SMOTE filter was used to balance the dataset^67^. The performance of the models used Area Under the Receiver Operating Characteristics (AUROC) metrics^68^, and 3-fold cross-validation (CV) method.

The best model to be used for predictions was chosen using criteria such as mean AUROC, standard deviation (SD) of AUROC, and the number of features. All the results obtained can be reproduced by using the scripts at https://github.com/muntisa/neural-networks-for-breast-cancer-proteins. The scaler, selected features and the best model were saved as files too. These are used to make predictions with another notebook for any new data (see 2-Predictions-BreastCancerPeptides.ipynb). We used these automatic scripts to predict the breast cancer activity for a 4,504 external proteins by using their molecular descriptors: 1,232 CIPs, 1,903 MDPs, and 1,369 RBPs.

After the screening of the 4,504 external proteins through the machine-learning model, complementary analyses were done to compare the amount of genomic alterations between BC related proteins (prediction 1) and BC non-related proteins (prediction 0). Firstly, we selected the study ‘Breast Invasive Carcinoma (TCGA, PanCancer Atlas)’ from the cBioPortal (https://www.cbioportal.org/)^69,70^, then, we downloaded and analyzed a matrix of CNAs (amplifications and deep deletions), putative mutations (inframe, truncating and missense), mRNA alterations (mRNA high and mRNA down), and protein alterations (high and low expression) related to the 4,504 proteins queried in a cohort of 1,066 individuals according to the Pan-Cancer Atlas^3,11,12,22^. Lastly, a Mann-Whitney U test was performed to obtain significant differences (p < 0.001) on the amount of genomic alterations between CIPs related and non-related to BC, MDPs related and non-related to BC, and RBPs related and non-related to BC.

## Results and Discussion

The current work proposes innovative classification models to predict new breast cancer proteins by using 6 sets of protein sequence descriptors calculated with Rcpi: AC, DC, TC, APAAC, MB and Mix. Python was used to build 13 types of machine-learning classifiers (NB, KNN, LDA, SVM linear, SVM, LR, MLP, DT, RF, XGB, GB, AdaB and Bagging), univariate filter as feature selection method, and PCA transformation of features. All the models used AUROC (mean values using 3-fold CV) to quantify the classification performance. Details about feature selection methods and parameters of machine-learning classifiers are included in the Supplementary_ML_Details.pdf.

**Figure 2:**
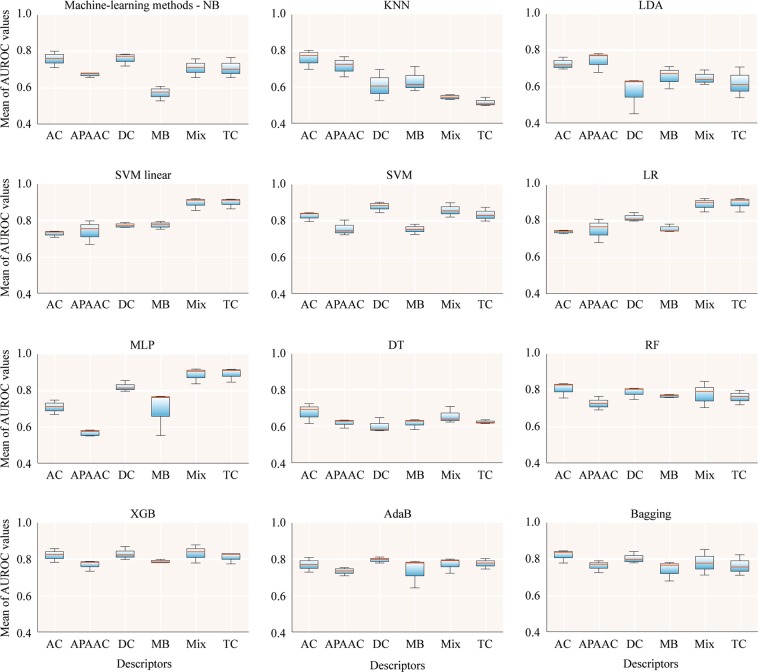
Mean AUROC of classifiers for breast cancer proteins using all features. NB, Gaussian Naive Bayes; KNN, k-nearest neighbors algorithm; LDA, linear discriminant analysis; SVM linear, support vector machine linear; LR, logistic regression; MLP, multilayer perceptron; DT, decision tree; RF, random forest; XGB, XGBoost; AdaB, AdaBoost classifier; Bagging, Bagging classifier; AC, amino acid composition; APAAC, amphiphilic pseudo-amino acid composition; DC, di-amino acid composition; MB, Moreau-Broto autocorrelation; Mix, total descriptors; TC, tri-amino acid composition.

For the first models, we used the pool of features for the six sets of descriptors without any feature selection or dimension reduction with 12 machine-learning methods (Fig. 2). We can observe that with a big number of descriptors in TC and Mix (over 8000), it is possible to obtain mean AUROC values greater than 0.9 with SVM linear, LR, and MLP. Even with 20 AC descriptors and XGB it is possible to obtain a mean AUROC of 0.857. But we tried to improve this performance and we applied univariate feature selection or PCA dimension reduction to diminish the number of inputs to a maximum of 300 features (due to the small number of instances).

**Figure 3:**
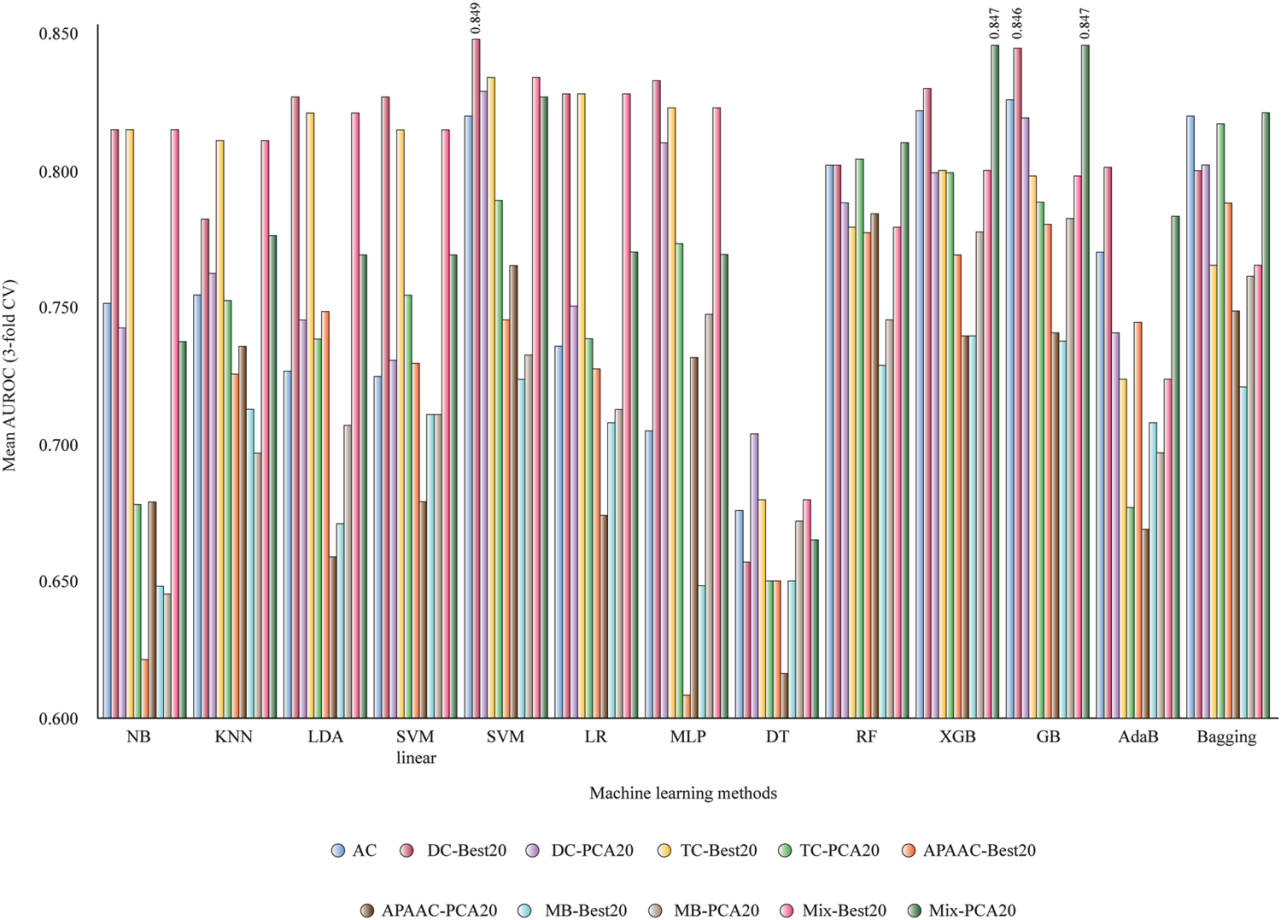
Mean AUROC values for classifiers obtained with 20 selected features (3-fold CV). NB, Gaussian Naive Bayes; KNN, k-nearest neighbors algorithm; LDA, linear discriminant analysis; SVM linear, support vector machine linear; LR, logistic regression; MLP, multilayer perceptron; DT, decision tree; RF, random forest; XGB, XGBoost; AdaB, AdaBoost classifier; Bagging, Bagging classifier; AC, amino acid composition; APAAC, amphiphilic pseudo-amino acid composition; DC, di-amino acid composition; MB, Moreau-Broto autocorrelation; Mix, total descriptors; TC, tri-amino acid composition.

Therefore, we selected models based on 20, 100, 200, and 300 features (see 1-ML-BreastCancerPeptides.ipynb). Figure 3 presents mean AUROC values for classifiers based on only 20 features: AC, DS-Best20, DC-PCA20, TC-Best20, TC-PCA20, APAAC-Best20, APAAC-PCA20, MB-Best20, MB-PCA20, Mix-Best20 and Mix-PCA20 (Best = univariate filter, PCA = feature transformation). DS-Best20 with only 20 di-amino acid composition descriptors and Mix-Best20 with a mixture of descriptors are able to offer mean AUROC values over 0.84 with non-linear SVM, XGB and GB. Additional results could be found in Supplementary Table 6.

**Figure 4:**
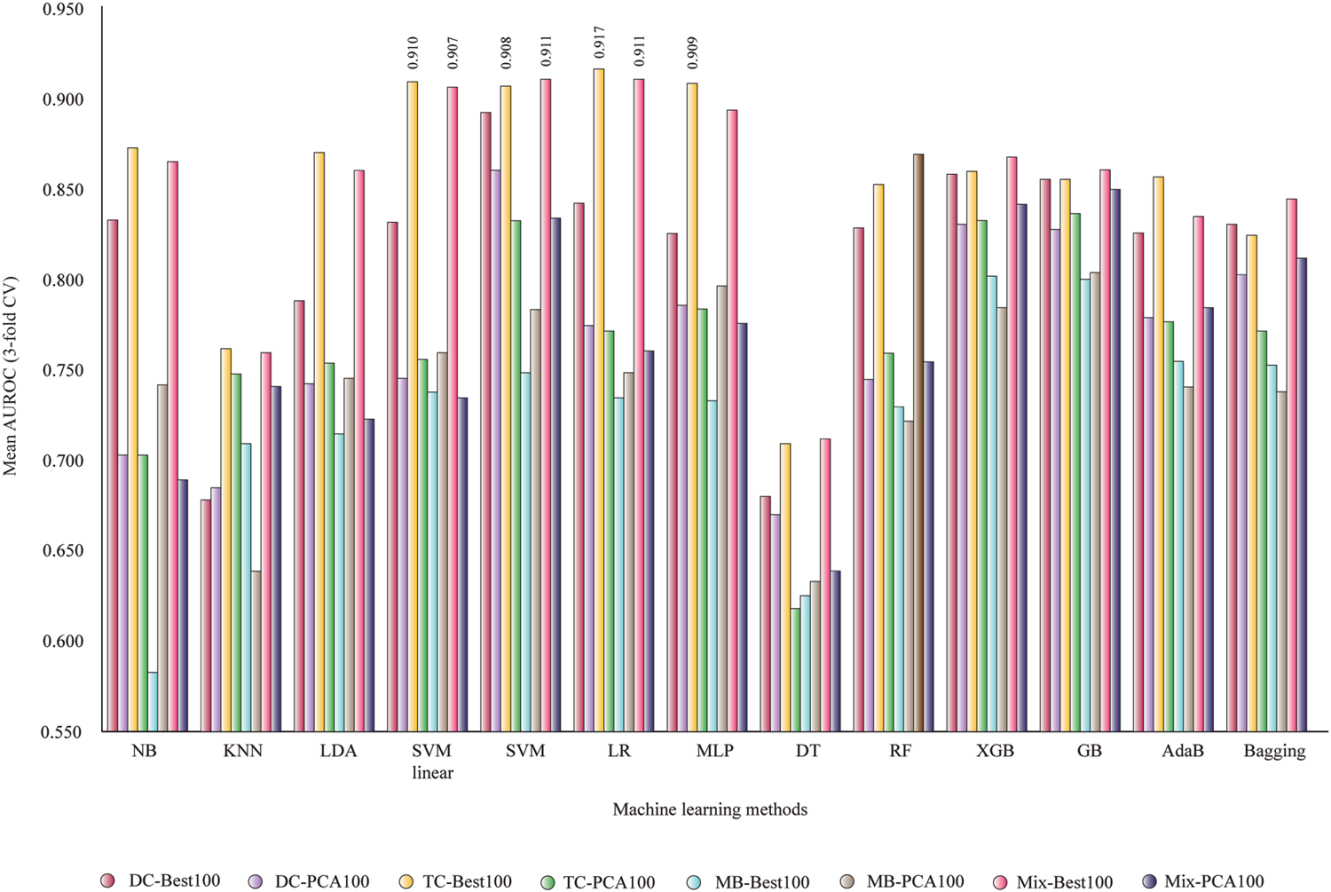
Mean AUROC for classifiers based on 100 selected features (3-fold CV). NB, Gaussian Naive Bayes; KNN, k-nearest neighbors algorithm; LDA, linear discriminant analysis; SVM linear, support vector machine linear; LR, logistic regression; MLP, multilayer perceptron; DT, decision tree; RF, random forest; XGB, XGBoost; AdaB, AdaBoost classifier; Bagging, Bagging classifier; DC, di-amino acid composition; MB, Moreau-Broto autocorrelation; Mix, total descriptors; TC, tri-amino acid composition.

If the number of features increased to 100 (5 times from 20), better AUROC values are obtained in Fig. 4: DC-Best100, DC-PCA100, TC-Best100, TC-PCA100, MB-Best100, MB-PCA100, Mix-Best100, and Mix-PCA100. Two sets of descriptors with four machine-learning methods are able to provide mean AUROC values greater than 0.9: TC-Best100 and Mix-Best100 with SVM linear, non-linear SVM, LR and MLP. Thus, LR and TC-Best100 (100 descriptors of tri-amino acid composition) generate a classifier with mean AUROC of 0.917. The increasing of AUROC values is important from 20 to 100 best descriptors. In the next step, the number of selected descriptors was increased to 200. The PCA transformed sets using the same number of components, as the selected features are not able to provide similar classification performance.

**Figure 5:**
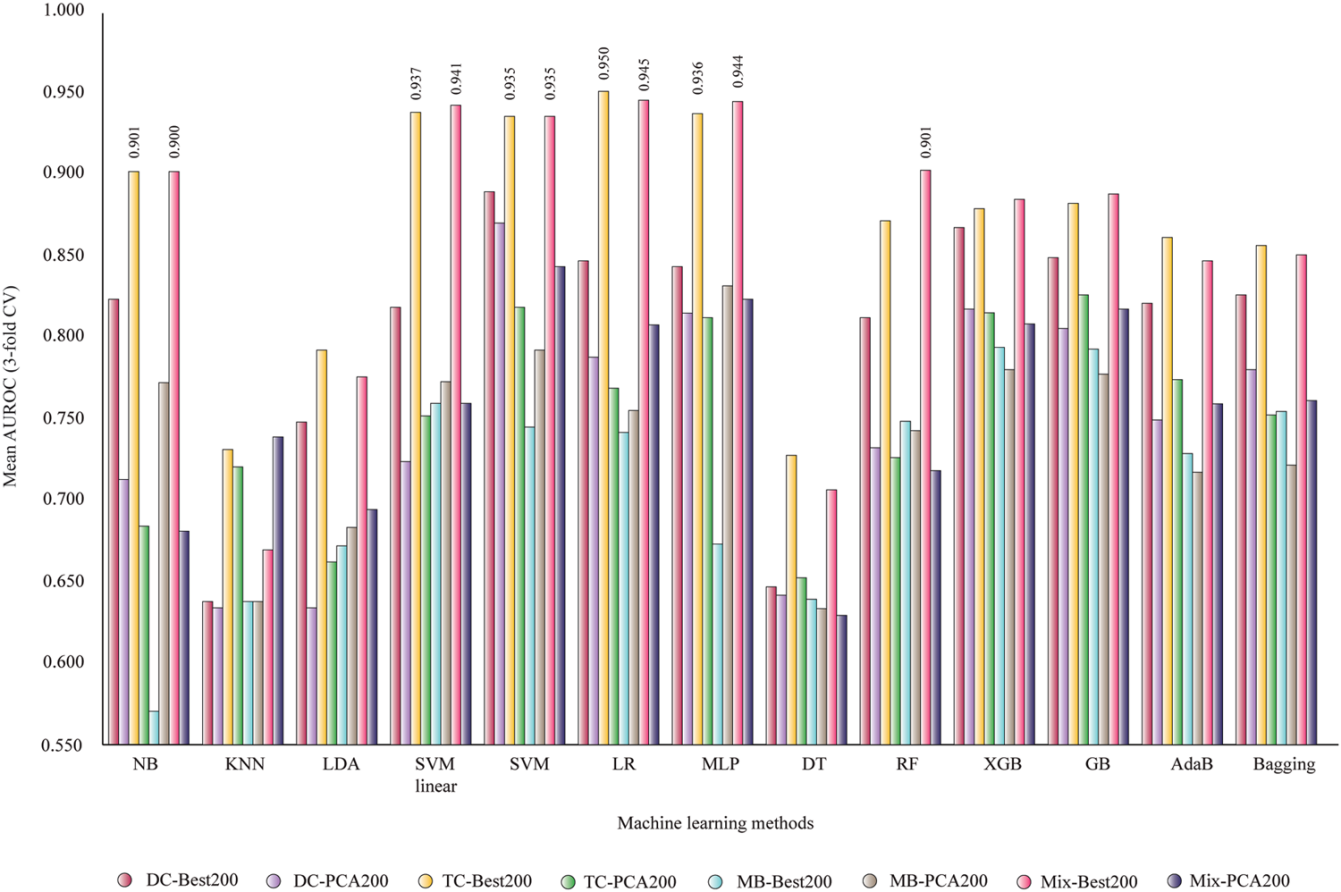
Mean AUROC of classifiers based on 200 selected features (3-fold CV). NB, Gaussian Naive Bayes; KNN, k-nearest neighbors algorithm; LDA, linear discriminant analysis; SVM linear, support vector machine linear; LR, logistic regression; MLP, multilayer perceptron; DT, decision tree; RF, random forest; XGB, XGBoost; AdaB, AdaBoost classifier; Bagging, Bagging classifier; DC, di-amino acid composition; MB, Moreau-Broto autocorrelation; Mix, total descriptors; TC, tri-amino acid composition.

Figure 5 presents the AUROC values for classifiers based on 200 selected features (a double number of inputs from 100): DC-Best200, DC-PCA200, TC-Best200, TC-PCA200, MB-Best200, MB-PCA200, Mix-Best200, and Mix-PCA200. We can observe that the same TC and Mix-based sets are providing mean AUROC values between 0.90 and 0.95 with five machine-learning methods: NB, SVM linear, LR, MLP, and RF. The maximum mean AUROC value was 0.950 using TC-Best200 and the simple linear LR method.

**Figure 6:**
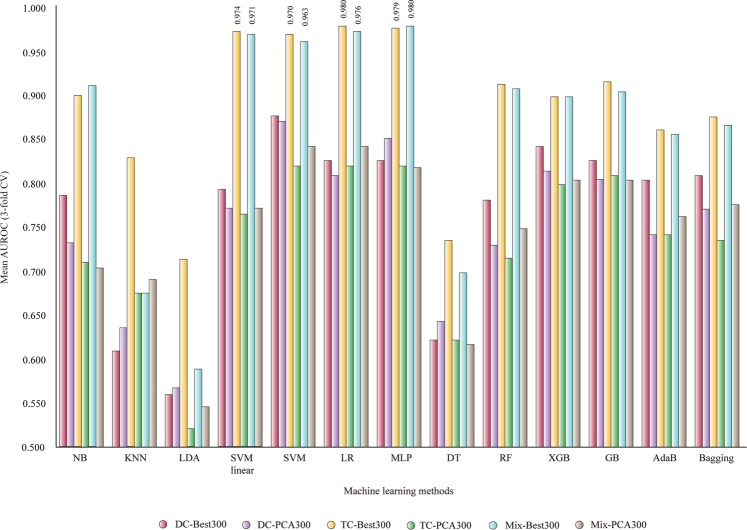
Mean AUROC of classifiers based on 300 selected features (3-fold CV). NB, Gaussian Naive Bayes; KNN, k-nearest neighbors algorithm; LDA, linear discriminant analysis; SVM linear, support vector machine linear; LR, logistic regression; MLP, multilayer perceptron; DT, decision tree; RF, random forest; XGB, XGBoost; AdaB, AdaBoost classifier; Bagging, Bagging classifier; DC, di-amino acid composition; MB, Moreau-Broto autocorrelation; Mix, total descriptors; TC, tri-amino acid composition.

In Fig. 6 the AUROC values for classifiers based on 300 selected features are presented: DC-Best300, DC-PCA300, TC-Best300, TC-PCA300, Mix-Best300, and Mix-PCA300. With 300 features, it is possible to provide more accurate classifier for BC proteins. The same TC and Mix subsets can generate classifiers with mean AUROC from 0.963 to 0.980 using SVM linear, SVM, LR and MLP.

The best AUROC of 0.980 ± 0.0037 was obtained with MLP and Mix-Best300. The same AUROC value was generated by TC-Best300 and LR but with a double SD of 0.0077. In the best model with the mixed descriptors, between the 300 descriptors, seven DC (LR, QI, NK, EM, QM, MM and EY) and two APAAC descriptors (Pc1.N and Pc1.M) were selected for BC function. The rest is TC descriptors without any MP descriptor selected (see Supplementary Table 7). The accuracy of the best model was 0.936 ± 0.0056. No methodology is perfect, and; therefore, our method/model has few weak sports: a) our dataset could be bigger: more examples/instances mean more accurate models. We were limited by the available database data; b) the best model has a relatively high number of descriptors: a model should use the minimum number of features because of simplicity, model explanation power, and to not overfit the dataset; c) our best model is an MLP with 300 descriptors and AUROC of 0.98, but in Figs. 3–6 we showed other different models obtained with other machine-learning methods, based on a smaller number of features. Thus, we can observe that it is possible to obtain a prediction model with an AUROC > 0.84 with only 20 descriptors. If the interest is the number of descriptors, the user could reproduce the models with the available notebooks and save any model; d) the best model is a black box such any neural network. If the explanation of the machine learning is the most important aspect, there are models with AUROC > 0.84 that could be explained better such as tree-based methods or linear models; e) our results could be improved by an extensive grid search of the hyperparameters of each machine-learning method. We did not consider this step because of the very high values of AUROC, which are fine for the purpose of this study.

In order to check if the best model is overfitted, we tried different CV folds (data splits) with the same MLP method (see CVs.ipynb for details). Thus, in the case of 5-fold CV, the mean AUROC was 0.9874 ± 0.0129 and the mean ACC was 0.9464 ± 0.0135. By increasing the number of folds to 10, the statistics showed a mean AUROC of 0.9831 ± 0.0158, and a mean ACC of 0.9401 ± 0.0226. All the models are saved into folder *best_classifier*. Therefore, we can conclude that the performance of the best model slightly increases with increased SD values. If these statistics are not fine for a specific application, it is possible to choose a different model based on 20 descriptors but with statistics greater than 0.80.

The 4,504 external proteins (1,903 without repetition) were transformed into the molecular descriptors of the best model and were used to predict the breast cancer activity (see 2-Predictions-BreastCancerPeptides.ipynb): 1,232 CIPs, 1,903 MDPs and 1,369 RBPs. Thus, all these proteins were transformed into 300 selected descriptors of a Mix-300 set and were used with the saved MLP classifier. As a result, 608 cancer immunotherapy proteins, 971 metastasis driver proteins and 757 RNA binding proteins were predicted to be related to breast cancer (Supplementary Tables 3 to 5).

### Cancer immunotherapy proteins

These proteins have a promising projection in clinical oncology due to successful long-term durable responses in advanced stages and metastasis. Similarly, cancer immunotherapy sparked tremendous interest in clinical, basic and translational science^71^. The 10 cancer immunotherapy proteins best related to BC, according to our machine-learning predictions, were RPS27, SUPT4H1, CLPSL2, POLR2K, RPL38, AKT3, CDK3, RPS20, RASL11A, and UNTD1 (Supplementary Table 3). For instance, Atsuta *et al*. determined that RPS27 is a tumor associated antigen in BC patients^72^.

**Figure 7:**
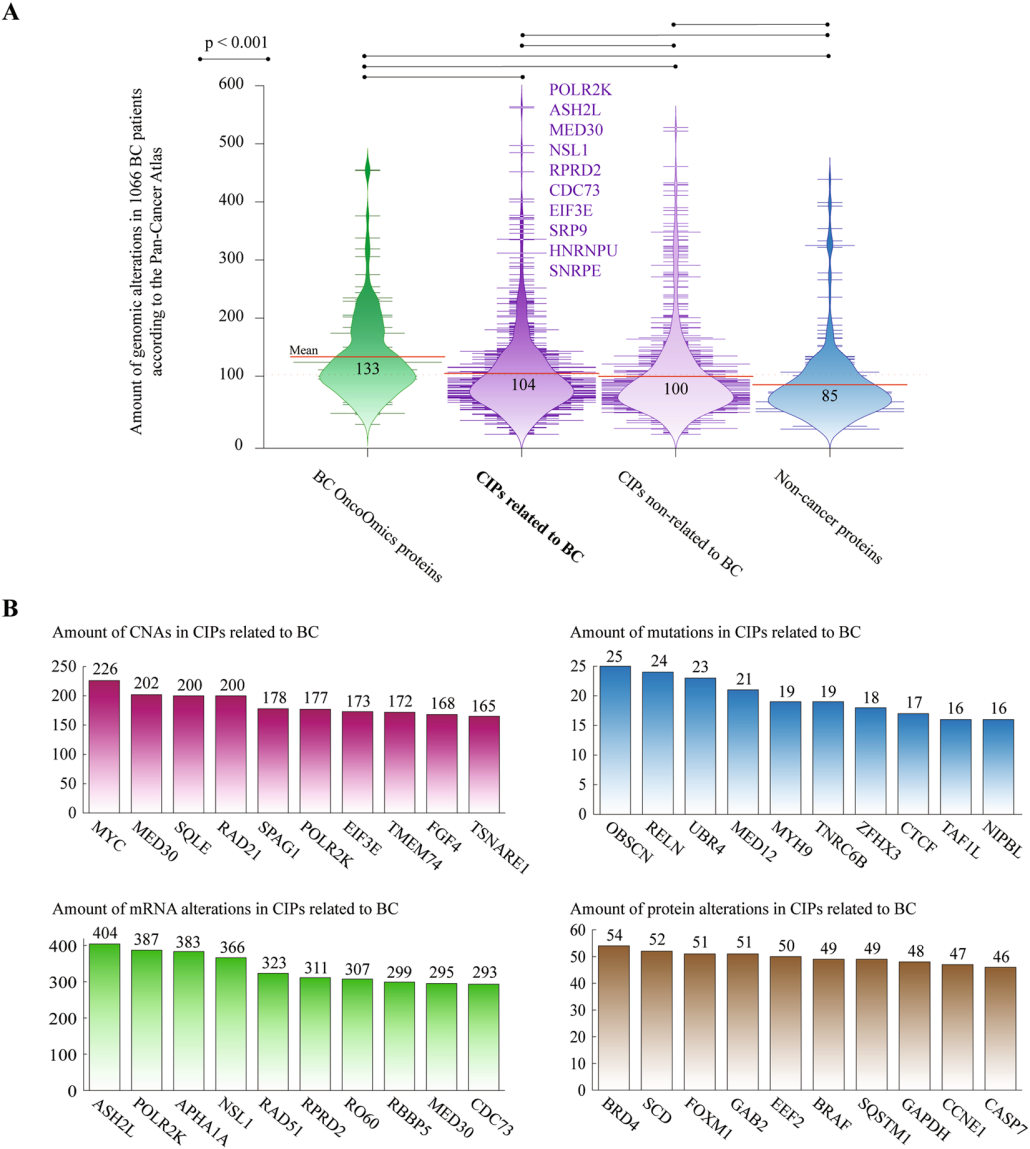
Cancer immunotherapy proteins (CIPs). (**A**) Bean plots comparing the amount (mean) of genomic alterations in 1066 patients between OncoOmics BC essential proteins, CIPs related to breast cancer, CIPs non-related to breast cancer, and non-cancer proteins according to the Pan-Cancer Atlas. (**B**) Ranking of the CIPs with the highest number of copy number alterations (CNAs), mutations, mRNA alterations, and protein alterations.

The development of cutting-edge technologies focused on the analysis of genomic alterations in cancer patients has allowed finding novel driver genes and therapeutic targets^73^. Hence, we performed an analysis to compare the amount of genomic alterations of the cancer immunotherapy proteins best related to breast cancer, according to the Pan-Cancer Atlas^3,11,12,22^. Figure 7A compares the amount of genomic alterations in a cohort of 1,066 patients between the OncoOmics BC essential proteins (mean of 133), CIPs related to BC (104), CIPs non-related to BC (100), and non-cancer proteins (85). As we can see, there was a significant difference (p < 0.001) of genomic alterations between CIPs related and non-related to BC after the Mann-Whitney U test. The top 10 CIPs related to BC and with the highest amount of genomic alterations were POLR2K, ASH2L, MED30, NSL1, RPRD2, CDC73, EIF3E, SRP9, HNRNPU and SNRPE (Supplementary Table 8). Additionally, Fig. 7B shows the most altered cancer immunotherapy proteins per genomic alteration type. MYC, OBSCN, ASH2L and BRD4 carried the highest number of CNAs, mutations, mRNA alterations and protein alterations, respectively.

### Metastasis driver proteins

Metastasis, often preceded or accompanied by therapeutic resistance, is the most lethal and insidious aspect of cancer. Due to treatment pressure, tumor evolution or mitochondria dysfunction, genomic alterations of metastatic tumors can differ substantially from primary tumors^74–76^. To date, the molecular and microenvironmental determinants of metastasis are largely unknown, as is the timing of systemic spread, hindering effective treatment and prevention efforts^66,77^. Integrated analysis of ‘omics’ data improves our understanding of BC metastasis. Moreover, these data would help us identify gene expression signature associated with metastasis in order to choose appropriate treatment strategies^78,79^. The 10 MDPs best related to BC, according to our machine-learning predictions, were S100A9, DDA1, TXN, PRNP, RPS27, S100A14, S100A7, MAPK1, AGR3 and NDUFA13 (Supplementary Table 4). For instance, Bergenfelz *et al*. suggested that S100A9 expressed in negative estrogen receptor and negative progesterone receptor breast cancers induces inflammatory cytokines and it is associated with an impaired overall survival^80^.

**Figure 8:**
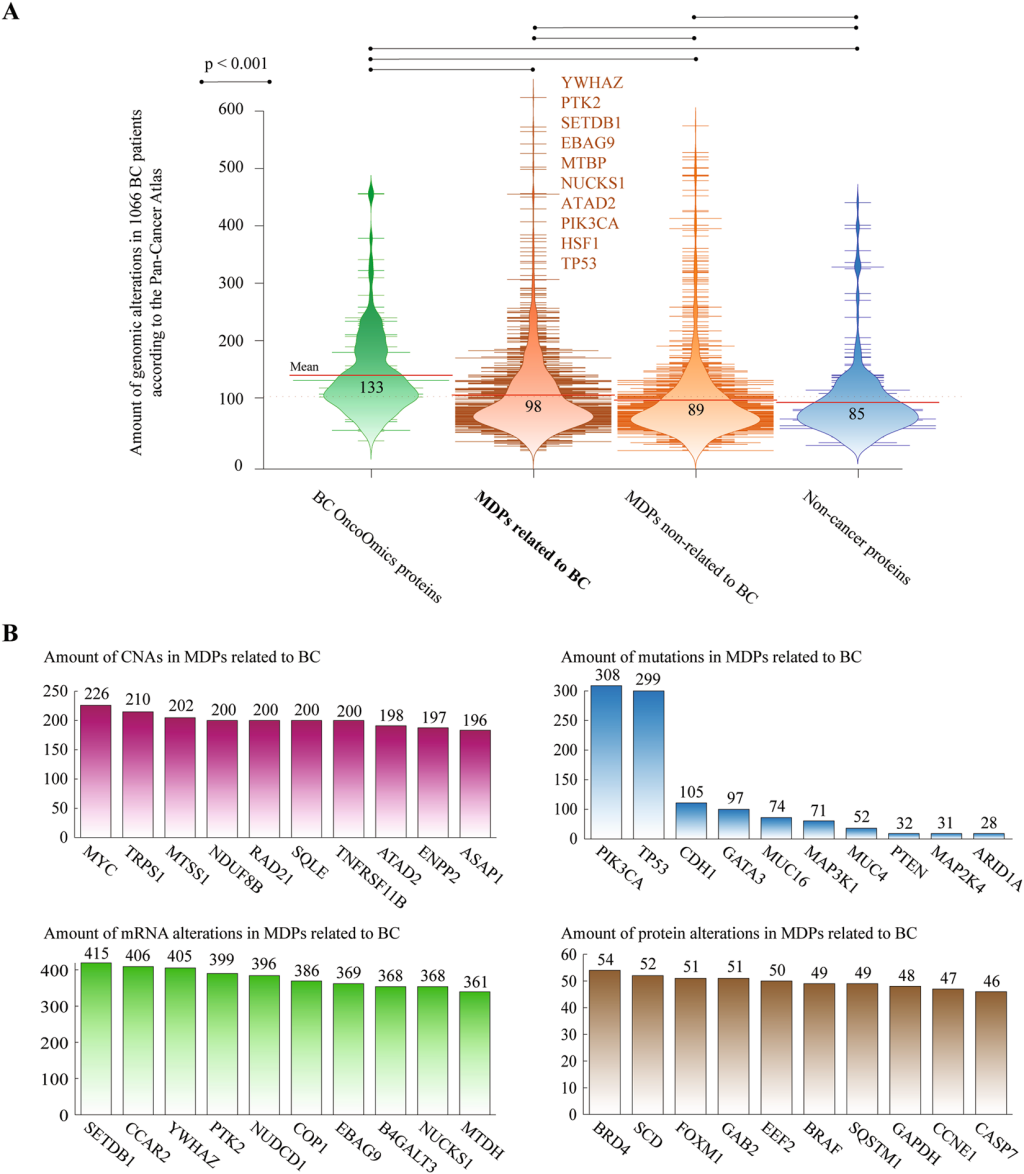
Metastasis driver proteins (MDPs). (**A**) Bean plots comparing the amount (mean) of genomic alterations in 1066 patients between OncoOmics BC essential proteins, MDPs related to breast cancer, MDPs non-related to breast cancer, and non-cancer proteins according to the Pan-Cancer Atlas. (**B**) Ranking of the MDPs with the highest number of copy number alterations (CNAs), mutations, mRNA alterations, and protein alterations.

Figure 8A shows bean plots comparing the amount of genomic alterations between the OncoOmics BC essential proteins (mean of 133), MDPs related to BC (98), MDPs non-related to BC (89) and non-cancer proteins (85). There was a significant difference (p < 0.001) of genomic alterations between MDPs related and non-related to BC after the Mann-Whitney U test. The top 10 MDPs related to BC and with the highest amount of genomic alterations were YWHAZ, PTK2, SETDB1, EBAG9, MTBP, NUCKS1, ATAD2, PIK3CA, HSF1 and TP53 (Supplementary Table 8). In addition, Fig. 8B shows the most altered metastasis driver proteins per genomic alteration type. MYC, PIK3CA, SETDB1 and BRD4 carried the highest number of CNAs, mutations, mRNA alterations and protein alterations, respectively.

### RNA-binding proteins

RNA biology is an under-investigated field of cancer even though pleiotropic changes in the transcriptome are key feature of cancer cell^81^. RBPs are able to control every aspect of RNA metabolism such as translation, splicing, stability, degradation of mRNA, nucleocytoplasmic transport, capping, and polyadenylation^81–85^. RBPs are emerging as critical modulators of BC and the prediction of relation with this complex disease through machine-learning methods provides a better understanding of new genomic targets and biomarkers. The 10 RBPs best related to BC, according to our machine-learning predictions were S100A9, TXN, RPS27L, RPS27, RPS27A, RPL38, MRPL54, PPAN, RPS20 and CSRP1 (Supplementary Table 5). For instance, Rodrigues *et al*. suggested that TXN is overexpressed in BC, and it is related to tumor grade, being a key element in redox homeostasis^86^.

**Figure 9:**
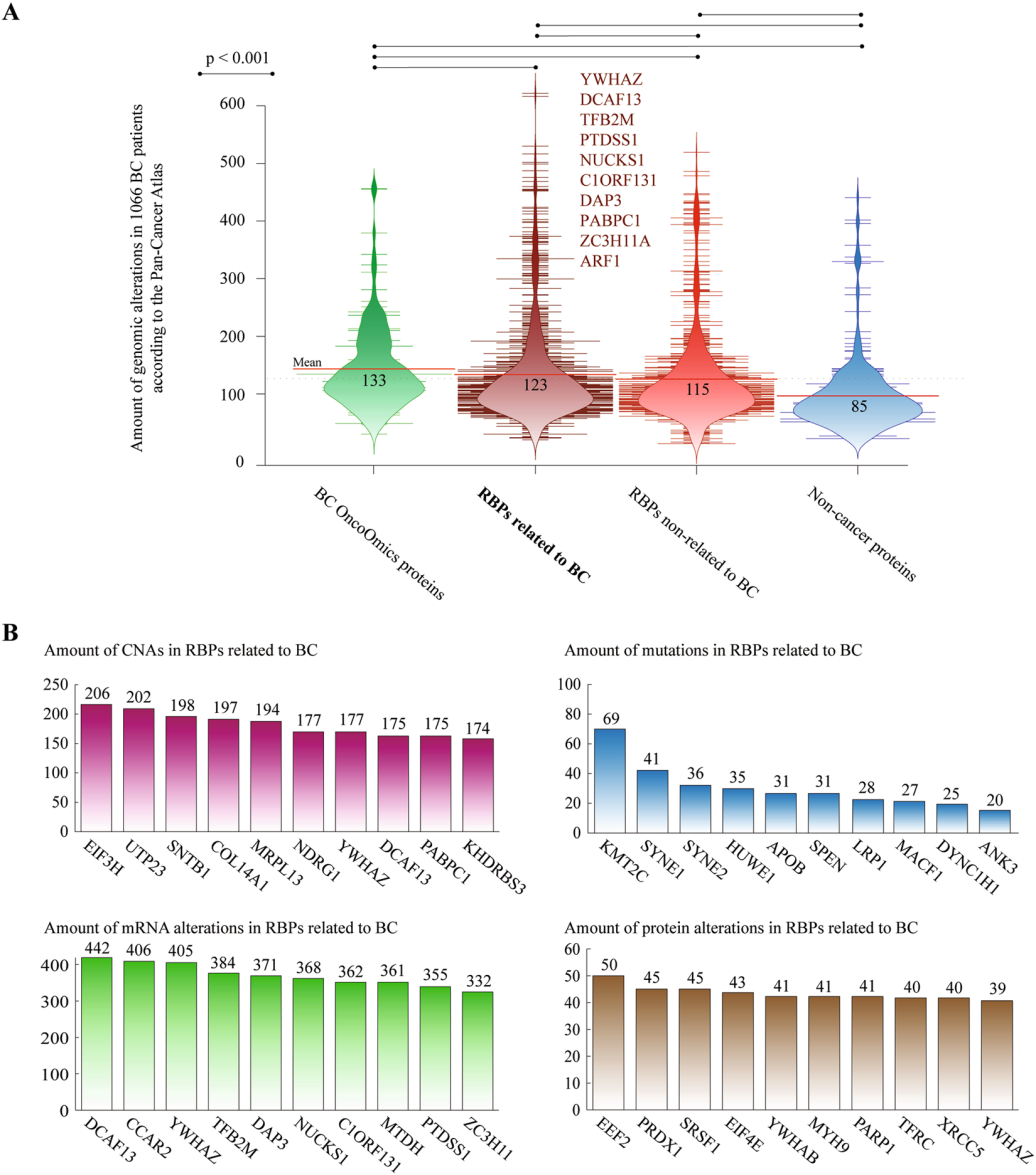
RNA-binding proteins (RBPs). (**A**) Bean plots comparing the amount (mean) of genomic alterations in 1066 patients between OncoOmics BC essential proteins, RBPs related to breast cancer, RBPs non-related to breast cancer, and non-cancer proteins according to the Pan-Cancer Atlas. (**B**) Ranking of the RBPs with the highest number of copy number alterations (CNAs), mutations, mRNA alterations, and protein alterations.

Figure 9A shows bean plots comparing the amount of genomic alterations between the OncoOmics BC essential proteins (mean of 133), RBPs related to BC (123), MDPs non-related to BC (115) and non-cancer proteins (85). There was a significant difference (p < 0.001) of genomic alterations between RBPs related and non-related to BC after the Mann-Whitney U test. The top 10 MDPs related to BC and with the highest amount of genomic alterations were YWHAZ, DCAF13, TFB2M, PTDSS1, NUCKS1, C1ORF131, DAP3, PABPC1, ZC3H11A and ARF1 (Supplementary Table 8). Additionally, Fig. 9B shows the most altered RNA-binding proteins per genomic alteration type. EIF3H, KMT2C, DCAF13 and EEF2 carried the highest number of CNAs, mutations, mRNA alterations and protein alterations, respectively.

Finally, the prediction of breast cancer proteins related to immunotherapy, metastasis and RNA-binding proteins is a key step to find novel therapeutic targets. For which we suggest multi-omics analyses of these predicted proteins using several databases focused on genomics, transcriptomics and proteomics in human tissues. Additionally, a future study will include the implementation of a web tool that will integrate the entire process predicting proteins with our saved model.

## Conclusions

The current study proposed better prediction models for breast cancer proteins using, as inputs, six sets of protein sequence descriptors from Rcpi and 13 machine-learning classifiers (with or without feature selection/dimension reduction of features). We choose, as the best classifier, the MLP classifier. As inputs, a mixture of 300 selected molecular descriptors has been used: DC, TC and APAAC. The model has a mean AUROC of 0.980 ± 0.0037 and a mean accuracy of 0.936 ± 0.0056 (3-fold cross-validation). 4,504 sequences of proteins related to cancer have been screened for breast cancer relation. Best predicted cancer immunotherapy proteins with BC were RPS27, SUPT4H1, CLPSL2, POLR2K and RPL38, and the most altered ones were POLR2K, ASH2L, MED30, NSL1 and RPRD2. Best predicted metastasis diver proteins with BC were S100A9, DDA1, TXN, PRNP and RPS27, and the most altered ones were YWHAZ, PTK2, SETDB1, EBAG9 and MTBP. Best predicted RNA-binding proteins with BC were S100A9, TXN, RPS27L, RPS27 and RPS27A, and the most altered ones were YWHAZ, DCAF13, TFB2M, PTDSS1 and NUCKS1. Finally, the association between the best-predicted BC proteins using powerful machine-learning methods and the amount of pathogenic genomic alterations in cancer immunotherapy proteins, metastasis driver proteins and RNA-binding proteins gives us candidate proteins that should be deeply studied to find novel therapeutic targets.

## Supplementary information

Supplementary Information.

Supplementary Dataset.

## Data Availability

All data generated during this study are included in this published article including its Supplementary Information files, and the scripts are available as free repository at https://github.com/muntisa/neural-networks-for-breast-cancer-proteins.
