# Supplementary material for: Prediction of breast cancer proteins involved in immunotherapy, metastasis, and RNA-binding using molecular descriptors and artificial neural networks
Source: Sci Rep. 2020 May 22;10:8515. doi: 10.1038/s41598-020-65584-y (PMC7244564; doi:10.1038/s41598-020-65584-y)
Supplement: Supplementary file 1 — Supplementary Information. [file 41598_2020_65584_MOESM1_ESM.pdf]

Prediction of breast cancer proteins involved in immunotherapy, metastasis, and RNA-binding using molecular descriptors and artificial neural networks

Andrés López-Cortés[1,2,3,†,*], Alejandro Cabrera-Andrade[2,4,5,†], José M. Vázquez-Naya[2,6,7], Alejandro Pazos[2,6,7], Humberto Gonzáles-Díaz[8,9], César Paz-y-Miño[1], Santiago Guerrero[1], Yunierkis Pérez-Castillo[4,10], Eduardo Tejera[4,11], Cristian R. Munteanu[2,6,7]

[1] Centro de Investigación Genética y Genómica, Facultad de Ciencias de la Salud Eugenio Espejo, Universidad UTE, Mariscal Sucre Avenue, Quito 170129, Ecuador

[2] RNASA-IMEDIR, Computer Science Faculty, University of Coruna, Coruna 15071, Spain

[3] Red Latinoamericana de Implementación y Validación de Guías Clínicas Farmacogenómicas (RELIVAF-CYTED)

[4] Grupo de Bio-Quimioinformática, Universidad de Las Américas, Avenue de los Granados, Quito 170125, Ecuador

[5] Carrera de Enfermería, Facultad de Ciencias de la Salud, Universidad de Las Américas, Avenue de los Granados, Quito 170125, Ecuador

[6] Centro de Investigación en Tecnologías de la Información y las Comunicaciones (CITIC), Campus de Elviña s/n 15071 A Coruña, Spain

[7] Biomedical Research Institute of A Coruña (INIBIC), University Hospital Complex of A Coruña (CHUAC), 15006, A Coruña, Spain

[8] Department of Organic Chemistry II, University of the Basque Country UPV/EHU, Leioa 48940, Biscay, Spain

[9] IKERBASQUE, Basque Foundation for Science, Bilbao 48011, Biscay, Spain

[10] Escuela de Ciencias Físicas y Matemáticas, Universidad de Las Américas, Avenue de los Granados, Quito 170125, Ecuador

[11] Facultad de Ingeniería y Ciencias Agropecuarias, Universidad de Las Américas, Avenue de los Granados, Quito 170125, Ecuador

[†] **These authors contributed equally to the study**

*** Correspondence:**

Andrés López-Cortés, MSc.

Centro de Investigación Genética y Genómica, Facultad de Ciencias de la Salud Eugenio Espejo, Universidad UTE, Mariscal Sucre Avenue, Quito 170129, Ecuador.

e-mail: aalc84@gmail.com

All the following details could be found in *1-ML-BreastCancerPeptides.ipynb* and *myFunctions.py* files:

https://github.com/muntisa/neural-networks-for-breast-cancer-proteins

Feature selection

The final dataset with selected descriptors and balanced dataset was saved as *Mix_BreastCancer.ds_bal.csv* in subfolder *best_classifier*. This is the list of the 300 selected features using Univariate Feature Selection method = SelectKBest(chi2, k=300):

'MN', 'LG', 'QI', 'NK', 'EM', 'QM', 'MM', 'EY', 'FAA', 'FNA', 'PNA', 'MDA', 'YHA', 'YKA', 'WFA', 'GPA', 'NT
A', 'EYA', 'PAR', 'QDR', 'KER', 'SQR', 'QGR', 'LLR', 'HKR', 'TKR', 'TMR', 'YMR', 'MFR', 'EAN', 'HAN', 'MR
N', 'SNN', 'EDN', 'QCN', 'QQN', 'GQN', 'PGN', 'IHN', 'NKN', 'HKN', 'LKN', 'AMN', 'TMN', 'VMN', 'MPN', 'P
SN', 'YTN', 'KWN', 'PWN', 'EYN', 'PYN', 'LVN', 'PVN', 'SVN', 'VAD', 'HRD', 'IND', 'PDD', 'IQD', 'NHD', 'Y
HD', 'NID', 'HFD', 'ITD', 'RYD', 'IYD', 'QRC', 'DNC', 'SNC', 'MDC', 'AQC', 'CGC', 'MGC', 'VHC', 'CKC', 'IK
C', 'SKC', 'MMC', 'PFC', 'MPC', 'MVC', 'FVC', 'FDE', 'YDE', 'SQE', 'TQE', 'RHE', 'MHE', 'HIE', 'FKE', 'EME',
'QME', 'LME', 'MME', 'VME', 'SFE', 'DAQ', 'TNQ', 'IDQ', 'DCQ', 'KCQ', 'GLQ', 'FKQ', 'AMQ', 'CMQ', 'VPQ',
'PSQ', 'IWQ', 'YWQ', 'CYQ', 'IAG', 'PAG', 'MNG', 'VGG', 'ALG', 'FLG', 'VLG', 'WMG', 'DFG', 'PFG', 'CTG', '
FWG', 'PWG', 'TVG', 'FAH', 'INH', 'PCH', 'VQH', 'CGH', 'CKH', 'IKH', 'SKH', 'MFH', 'WFH', 'HTH', 'RWH', '
FWH', 'GYH', 'RVH', 'KRI', 'MRI', 'AQI', 'LQI', 'NHI', 'NII', 'QII', 'CKI', 'YKI', 'IPI', 'WSI', 'EYI', 'LYI', 'MVI',
'RDL', 'TCL', 'CQL', 'HQL', 'MQL', 'GGL', 'LGL', 'HLL', 'HML', 'QWL', 'EYL', 'GVL', 'IVL', 'HAK', 'KNK', '
VNK', 'SCK', 'TQK', 'KHK', 'VHK', 'QIK', 'PIK', 'DLK', 'MMK', 'EPK', 'HTK', 'CWK', 'DVK', 'WAM', 'HRM',
'QNM', 'PNM', 'VDM', 'AEM', 'EEM', 'QQM', 'QGM', 'RIM', 'CIM', 'QLM', 'ILM', 'HKM', 'AMM', 'NMM', 'C
FM', 'NTM', 'TTM', 'VTM', 'CWM', 'EWM', 'FYM', 'TAF', 'YCF', 'AEF', 'RQF', 'VGF', 'PKF', 'AMF', 'YFF', 'H
TF', 'GYF', 'DAP', 'MNP', 'SNP', 'RGP', 'QGP', 'PGP', 'VGP', 'WLP', 'DKP', 'IKP', 'LKP', 'CMP', 'IFP', 'QTP', '
KTP', 'IWP', 'MNS', 'KQS', 'WGS', 'CHS', 'MMS', 'TMS', 'LNT', 'DCT', 'CQT', 'PQT', 'FHT', 'IIT', 'PIT', 'GLT',
'MLT', 'VLT', 'MKT', 'WKT', 'TMT', 'IFT', 'MPT', 'EWT', 'QWT', 'KWT', 'EYT', 'GAW', 'LNW', 'ADW', 'HC
W', 'NEW', 'EEW', 'YEW', 'DQW', 'QHW', 'GIW', 'HIW', 'LLW', 'IKW', 'VKW', 'FPW', 'RTW', 'VTW', 'WYW
', 'DVW', 'SVW', 'NDY', 'PDY', 'ECY', 'GCY', 'MEY', 'TEY', 'EIY', 'KIY', 'PIY', 'EMY', 'MPY', 'TPY', 'HWY',
'KNV', 'VNV', 'NDV', 'KCV', 'GHV', 'GLV', 'MMV', 'VFV', 'IYV', 'Pc1.N', 'Pc1.M'

This method computes chi-squared stats between each non-negative feature and the class:
https://scikit-learn.org/stable/modules/generated/sklearn.feature_selection.chi2.html#sklearn.feature_selection
.chi2

Machine Learning classifiers

The list with all classifiers and the parameters used for calculations are presented below. The rest of the param eters are the default ones.

```
classifiers = [GaussianNB(),
            KNeighborsClassifier(3),
            LinearDiscriminantAnalysis(solver='svd',priors=priors)
            SVC(kernel="linear",random_state=seed,gamma='scale',class_weight=class_weights),
            SVC(kernel = 'rbf', random_state=seed,gamma='scale',class_weight=class_weights),
            LogisticRegression(solver='lbfgs',random_state=seed,class_weight=class_weights),
            MLPClassifier(hidden_layer_sizes= (20), random_state = seed, max_iter=50000, shuffle=False),
            DecisionTreeClassifier(random_state = seed,class_weight=class_weights),
            RandomForestClassifier(n_jobs=-1,random_state=seed,class_weight=class_weights),
            XGBClassifier(n_jobs=-1,seed=seed,scale_pos_weight= class_weights[0]/class_weights[1]),
            GradientBoostingClassifier(random_state=seed),
            AdaBoostClassifier(random_state = seed),
            BaggingClassifier(random_state=seed)
            ]
```

class_weights = {0: 1, 1: 1} and priors = [(class_weights[0]/(class_weights[0]+class_weights[1])), (class_wei ghts[1]/(class_weights[0]+class_weights[1]))]. These parameters are used to keep into account unbalanced dat asets.

The complete list with all the parameters (visible and default values) are presented below:

```
GaussianNB(priors=None, var_smoothing=1e-09)
KNeighborsClassifier(algorithm='auto', leaf_size=30, metric='minkowski',
        metric_params=None, n_jobs=None, n_neighbors=3, p=2,
        weights='uniform')
LinearDiscriminantAnalysis(n_components=None, priors=[0.5, 0.5],
          shrinkage=None, solver='svd', store_covariance=False,
          tol=0.0001)
SVC(C=1.0, cache_size=200, class_weight={0: 1, 1: 1}, coef0=0.0,
  decision_function_shape='ovr', degree=3, gamma='scale', kernel='linear',
  max_iter=-1, probability=False, random_state=74, shrinking=True,
  tol=0.001, verbose=False)
SVC(C=1.0, cache_size=200, class_weight={0: 1, 1: 1}, coef0=0.0,
  decision_function_shape='ovr', degree=3, gamma='scale', kernel='rbf',
  max_iter=-1, probability=False, random_state=74, shrinking=True,
  tol=0.001, verbose=False)
LogisticRegression(C=1.0, class_weight={0: 1, 1: 1}, dual=False,
        fit_intercept=True, intercept_scaling=1, max_iter=100,
        multi_class='warn', n_jobs=None, penalty='l2', random_state=74,
        solver='lbfgs', tol=0.0001, verbose=0, warm_start=False)
MLPClassifier(activation='relu', alpha=0.0001, batch_size='auto', beta_1=0.9,
      beta_2=0.999, early_stopping=False, epsilon=1e-08,
      hidden_layer_sizes=20, learning_rate='constant',
      learning_rate_init=0.001, max_iter=50000, momentum=0.9,
      n_iter_no_change=10, nesterovs_momentum=True, power_t=0.5,
      random_state=74, shuffle=False, solver='adam', tol=0.0001,
      validation_fraction=0.1, verbose=False, warm_start=False)
DecisionTreeClassifier(class_weight={0: 1, 1: 1}, criterion='gini',
         max_depth=None, max_features=None, max_leaf_nodes=None,
         min_impurity_decrease=0.0, min_impurity_split=None,
```

```
            min_samples_leaf=1, min_samples_split=2,
            min_weight_fraction_leaf=0.0, presort=False, random_state=74,
            splitter='best')
RandomForestClassifier(bootstrap=True, class_weight={0: 1, 1: 1},
            criterion='gini', max_depth=None, max_features='auto',
            max_leaf_nodes=None, min_impurity_decrease=0.0,
            min_impurity_split=None, min_samples_leaf=1,
            min_samples_split=2, min_weight_fraction_leaf=0.0,
            n_estimators='warn', n_jobs=-1, oob_score=False,
            random_state=74, verbose=0, warm_start=False)
XGBClassifier(base_score=0.5, booster='gbtree', colsample_bylevel=1,
       colsample_bytree=1, gamma=0, learning_rate=0.1, max_delta_step=0,
       max_depth=3, min_child_weight=1, missing=None, n_estimators=100,
       n_jobs=-1, nthread=None, objective='binary:logistic',
       random_state=0, reg_alpha=0, reg_lambda=1, scale_pos_weight=1.0,
       seed=74, silent=True, subsample=1)
GradientBoostingClassifier(criterion='friedman_mse', init=None,
              learning_rate=0.1, loss='deviance', max_depth=3,
              max_features=None, max_leaf_nodes=None,
              min_impurity_decrease=0.0, min_impurity_split=None,
              min_samples_leaf=1, min_samples_split=2,
              min_weight_fraction_leaf=0.0, n_estimators=100,
              n_iter_no_change=None, presort='auto', random_state=74,
              subsample=1.0, tol=0.0001, validation_fraction=0.1,
              verbose=0, warm_start=False)
AdaBoostClassifier(algorithm='SAMME.R', base_estimator=None,
          learning_rate=1.0, n_estimators=50, random_state=74)
BaggingClassifier(base_estimator=None, bootstrap=True,
         bootstrap_features=False, max_features=1.0, max_samples=1.0,
         n_estimators=10, n_jobs=None, oob_score=False, random_state=74,
         verbose=0, warm_start=False)
```

MLP classifier (best model) has only 20 neurons in a single hidden layer and maximum 50000 iterations for t raining.
